# Cucurbitacin E as Inducer of Cell Death and Apoptosis in Human Oral Squamous Cell Carcinoma Cell Line SAS

**DOI:** 10.3390/ijms140817147

**Published:** 2013-08-20

**Authors:** Chao-Ming Hung, Chi-Chang Chang, Chen-Wei Lin, Shun-Yao Ko, Yi-Chiang Hsu

**Affiliations:** 1 Department of General Surgery, E-Da Hospital, I-Shou University, Kaohsiung 82445, Taiwan; 2 Department of Obstetrics and Gynecology, E-Da Hospital, I-Shou University, Kaohsiung 82445, Taiwan; E-Mail: p2696373@yahoo.com.tw; 3 Graduate Institute of Medical Science, College of Health Sciences, Chang Jung Christian University, Tainan 71101, Taiwan; E-Mails: bigetboy@hotmail.com (C.-W.L.); syko@mail.cjcu.edu.tw (S.-Y.K.); 4 Innovative Research Center of Medicine, College of Health Sciences, Chang Jung Christian University, Tainan 71101, Taiwan

**Keywords:** cucurbitacin E, human oral squamous cell carcinoma (OSCC), apoptosis, caspase

## Abstract

Human oral squamous cell carcinoma (OSCC) is a common form of malignant cancer, for which radiotherapy or chemotherapy are the main treatment methods. Cucurbitacin E (CuE) is a natural compound previously shown to be an antifeedant as well as a potent chemopreventive agent against several types of cancer. The present study investigates anti-proliferation (using MTT assay, CuE demonstrated cytotoxic activity against SAS cell with IC_50_ values at 3.69 μM) and induced apoptosis of human oral squamous cell carcinoma SAS cells after 24 h treatment with CuE. Mitochondrial membrane potential (MMP) and caspase activity were studied and our results indicate that CuE inhibits cell proliferation as well as the activation of apoptois in SAS cells. Both effects increased in proportion to the dosage of CuE and apoptosis was induced via mitochondria- and caspase-dependent pathways. CuE can induce cell death by a mechanism that is not dependent on apoptosis induction, and thus represents a promising anticancer agent for prevention and treatment of OSCC.

## 1. Introduction

Oral squamous cell carcinoma (OSCC) is a malignant tumor common commonly diagnosed oral cancer in Taiwan. The age of OSCC onset tends to be younger than that of other tumors, affecting most patients at approximately 30–50 years of age [1]. Although the etiologies underlying the development of OSCC are not fully understood, tobacco use, alcohol, and betel quid chewing are major risk factors believed to play important roles in the development of carcinogenesis [2]. Radiotherapy is the mainstay of treatment. However, the prognosis is poor for relapsed and refractory disease or metastatic even after therapeutic interventions with chemotherapeutic treatment [3].

Cucurbitacins are a group of tetracyclic triterpenes with medicinal properties derived from the climbing stem of *Cucumic melo L* [4]. They have been used extensively in traditional medicines throughout Asia and many cancer-preventive properties have previously been reported [5]. Interest in this herb has grown in recent years, due to its putative beneficial pharmacological effects as an anti-inflammatory [6] and anticancer agent [7,8]. There have also been indications that cucurbitacins may help in the prevention and treatment of oxidative damage as well as the suppression of specific inflammatory factors [9].

Cucurbitacin E (CuE, α-elaterin) is an active compound [10], previously shown to be a strong antifeedant with the ability to disrupt cellular actin [11] and cell adhesion. Recent reports have demonstrated that CuE has an inhibitory effect on cancer cell proliferation, actin polymerization, and permeability [12]. However, whether CuE inhibits OSCC growth remains unknown. Furthermore, the mechanism underlying the anti-cancer effect of CuE has yet to be identified.

This present study was initiated to investigate whether CuE contributes to the anti-proliferation and apoptosis of SAS cells. It is expected that these experiments will provide a scientific basis and technological support for further development of OSCC therapy.

## 2. Results and Discussion

### 2.1. CuE Inhibits Cell Survival/Proliferation of SAS Cells

**Figure 1:**
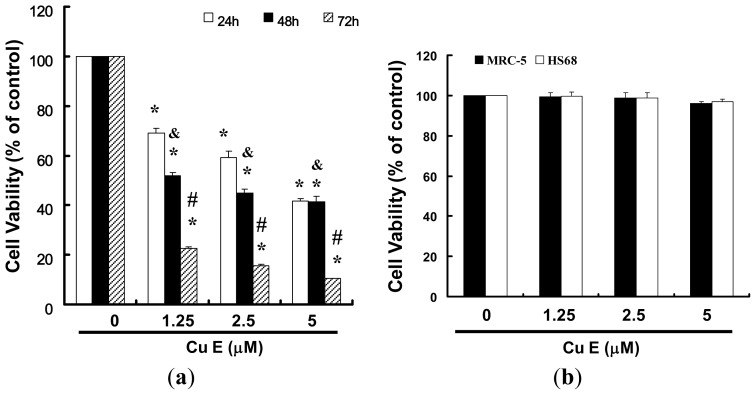
CuE mediates the survival of SAS and normal fibroblast (MRC-5; normal lung fibroblasts and HS68; normal skin fibroblasts) cells, thereby inhibiting the proliferation in SAS cells. (**a**) An *in vitro* study was initiated by treating the SAS cells with increasing doses of CuE (0, 1.25, 2.5 and 5 μM) for 24 to 72 h; and (**b**) MRC-5 and HS68 cells treated with CuE for 24 h. The survival of CuE-treated cells was then measured using the MTT method. Results were expressed as a percentage of control, which was considered 100%. All data were reported as the mean (±SEM) of at least three separate experiments. Statistical analysis was performed using a *t*-test, with significant differences determined at the level of *p < 0.05 *vs.* the control group (CuE: 0 μM), ^&^*p* < 0.05 *vs.* 24 h and ^#^*p* < 0.05 *vs.*48 h.

We hypothesized that CuE could mediate the survival of the OSCC cell line and thus inhibit their proliferation. To explore this anti-tumor activity of CuE against the SAS cells, an *in vitro* study was initiated by treatment of the SAS cells with increasing doses of CuE (0, 1.25, 2.5 and 5 μM) for 24 h. The proliferation of these CuE-treated cancer cells was then measured by the MTT method; the results summarized in Figure 1 indicate that the survival and proliferation of the SAS cells both decreased per increase of the dose of CuE added to the cell cultures, but not in the MRC-5 and HS68 cells (Figure 1b) which show a dose-dependent reduction (*y* = 8.15*x* + 19.903, *R*^2^ = 0.961). Moreover, CuE was noted to induce a morphological change in the SAS cells. A microscopic examination showed that following the exposure to CuE (2.5 μM) for 6 to 24 h, the cells displayed a remarkable change in their morphology and CuE induced the death of cancer cells, which formed a suspension in the medium (data not shown).

### 2.2. Apoptosis of SAS Cells Induced by CuE

**Figure 2:**
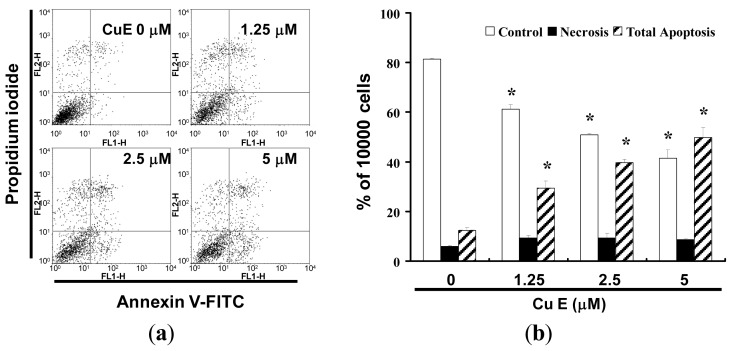
(**a**) Influence of CuE on apoptosis in SAS cells; and (**b**) Total apoptosis in SAS cells after 4 h of incubation with CuE. (*p < 0.05 *vs.* CuE 0 μM control group).

Detection between the intact cells, early apoptotic cells and late apoptotic cells or dead cells could be carried out with PI-annexin-V double staining; thus, we performed this assay to further explore cell apoptosis. To explore the potential role that CuE could play in the apoptosis of SAS cells, the ApopNexin FITC apoptosis detection kit has been used to identify the formation of apoptotic cells in the SAS cells after 6 h exposure to CuE. A typical set of results for the ApopNexin FITC apoptosis detection kit is illustrated in Figure 2a, in which the annexin V-FITC deposits are indicative of the positive existence of apoptotic cells. A dose-dependent increase in apoptosis was observed (*y* = 12.227*x* + 2.2467, *R*^2^ = 0.9819), that is: the higher the dose of CuE (1.25, 2.5 and 5 μM) used in the exposure, the greater the extent of apoptosis (Figure 2b). The increase of the percentages of apoptotic SAS cells was observed at all doses after treatment for 4 h. In 4 h, approximately 12.44 ± 0.92% of SAS cells were totally apoptotic (early apoptosis and late apoptosis) relative to cells in CuE 0 μM. The rate of apoptotic SAS cells increased to 29.38% ± 2.97% with 1.25 μM CuE treatments. When the concentrations of CuE increased to 2.5 and 5 μM, the percentages of total apoptotic CRC cells increased to 39.65% ± 1.34% and 49.77% ± 4.04% respectively. Taken together, the observations imply that the apoptosis of SAS cells is significantly elevated by CuE.

### 2.3. CuE Treatment Accumulated Sub G1 in SAS Cells

**Figure 3:**
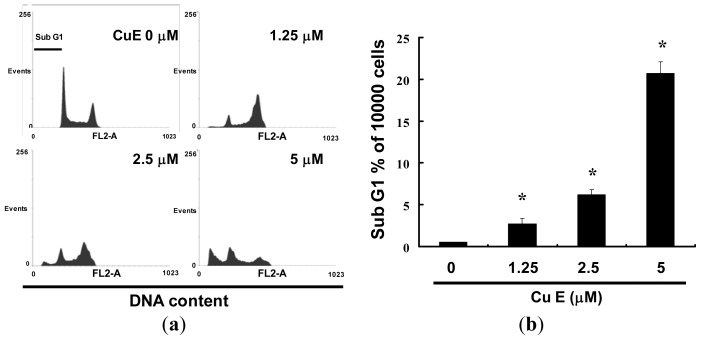
Influence of CuE on cell cycle progression/distribution in SAS cells: (**a**) Cell cycle analysis of SAS cells after being cultured with CuE for 24 h; and (**b**) CuE induced an increase in subG1 (%). Cells were stained with propidium iodide to analyze DNA content and quantified by flow cytometry. Symbol (*) in each group of bars indicates that the difference resulting from treatment with CuE 0 μM is statistically significant at *p* < 0.05.

Cell-cycle distribution of CuE-treated SAS cells was analyzed by flow cytometry, aiming to determine whether the inhibitory effect was due to cell-cycle arrest and apoptosis. Before being processed and analyzed, the cells were exposed to CuE for a total of 24 h. As shown in Figure 3a, the SAS cells exposed to CuE showed an increase in the number of cells in the subG0/G1 phase, as compared with that of the untreated cells. These results revealed that the CuE block of SAS cell proliferation caused a significant increase in the proportion of cells in the subG0/G1 phase. The results in Figure 3b suggest that the SAS cells accumulated in the sub G_0_/G_1_ phase (*y* = 12.227*x* + 2.2467, *R*^2^ = 0.8282) following the CuE treatment for 24 h. The observations could imply that the SAS cells have undergone apoptosis.

### 2.4. Apoptosis Induction by CuE in SAS Cells via Caspase 3 Activation

**Figure 4:**
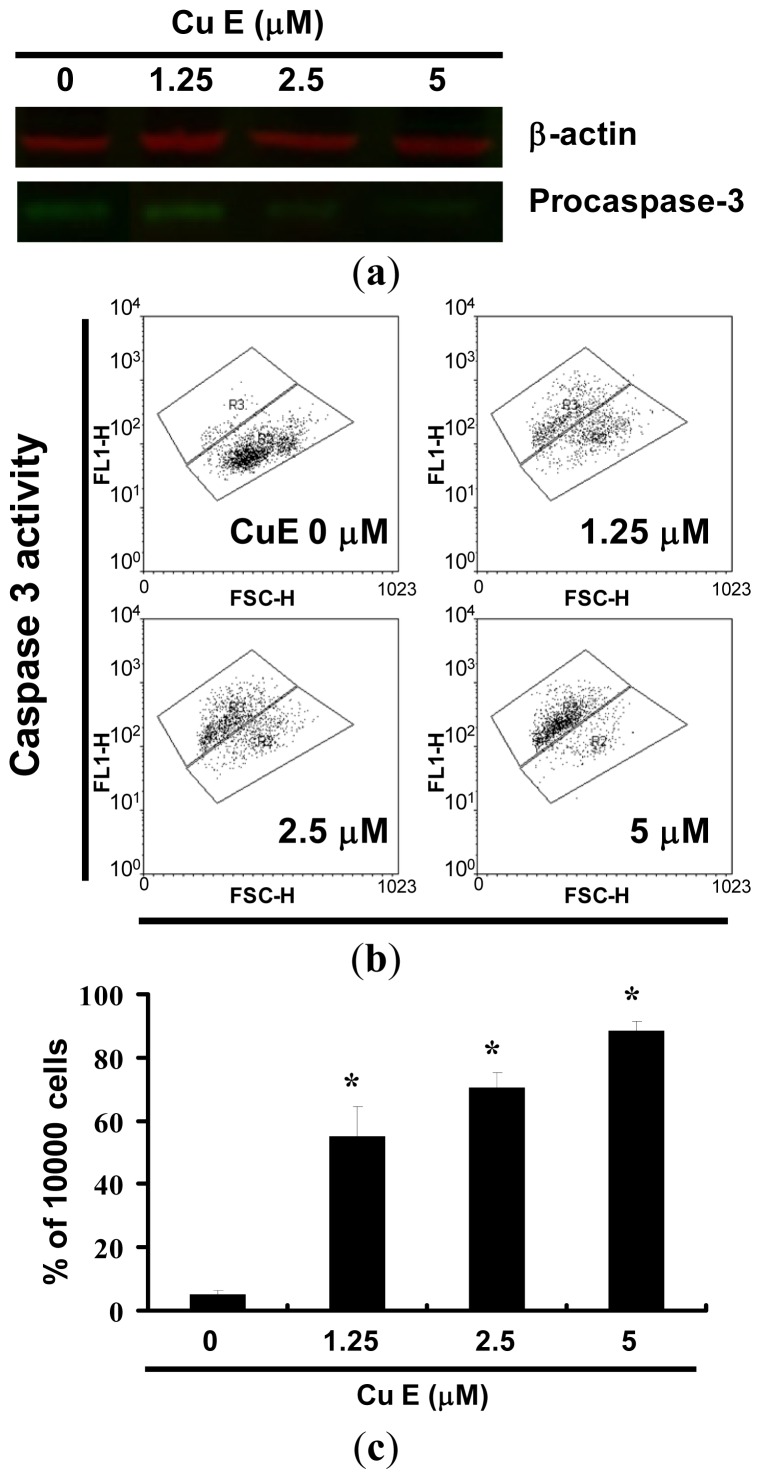
Caspase-3 activity in SAS cells by CuE 24 h treatment. (**a**) The cells were treated with CuE (0, 1.25, 2.5 and 5 μM) for 24 h and procaspase-3 proteins were subsequently detected by Western blot analysis; (**b**) Following treatment, the cells were harvested and labeled using FITC rabbit anti-active caspase-3. Activation was quantified by flow cytometry; and (**c**) Quantification by flow cytometry. All data was reported as the mean (±SEM) of at least three separate experiments.

Figure 4a shows the immunoblotting of cellular proteins from SAS cells treated with CuE showing the decrease of pro-caspase-3 after CuE incubation. Quantification of pro-caspase-3, through measurement of the relative band intensities, showed that pro-caspase-3 levels were significantly lower in cells incubated with CuE (data not shown). Caspase 3 activation at CuE concentrations of 1.25 to 5 μM is shown in Figure 4b. The results in Figure 4c suggest that the level of caspase 3 activation in SAS cells were elevated (*y* = 26.621*x* − 11.728, *R*^2^ = 0.9161) following CuE treatment for 24 h. However, the results summarized in Figures 2–4 indicate that CuE may mediate the survival of SAS cells. Thus, we hypothesize that the proliferation of these cells was inhibited by pathways other than apoptosis.

### 2.5. Assessment of Changes in Mitochondrial Membrane Potential

**Figure 5:**
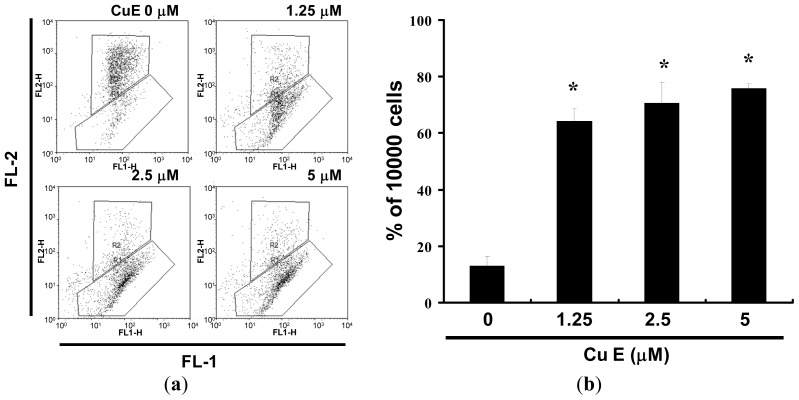
Reduction of mitochondrial membrane potential (MMP) in SAS cells by CuE, as determined by JC-1 staining and detected by flow cytometry: (**a**) MMP is shown to be significantly reduced in the SAS cells treated with CuE (0, 1.25, 2.5 and 5 M) by JC-1 staining; and (**b**) All the data shown are the mean (±SEM) of at least three independent experiments. The symbols (*) in each group of bars indicates that the difference resulting from treatment with CuE is statistically significant at *p* < 0.05 *vs.* CuE 0 μM.

The loss of mitochondrial membrane potential is a hallmark for apoptosis. It is an early event coinciding with caspase activation. In non-apoptotic cells, JC-1 exists as a monomer in the cytosol (green) and accumulates as aggregates in the mitochondria, which appear red. In apoptotic and necrotic cells, JC-1 exists in monomeric form and stains the cytosol green. Figure 5a shows typical FL-1/FL-2 dot plots for JC-1 staining SAS cells with and without apoptosis. CuE-free SAS cells are without apoptosis, which have red fluorescing J-aggregates. The green fluorescing monomers shown in the lower part indicate apoptotic cell lines (CuE 1.25, 2.5 and 5 μM treatment). Figure 5b shows the percentages of apoptotic SAS cells analyzed by flow cytometry in different CuE-treated groups. The x-fold increase of mitochondrial membrane potential lost was observed at all doses after treatment for 6 h. At 6 h, the folds increased to 5.36 ± 2.12 times with 1.25 μM CuE treatment. When the concentrations of CuE increased to 2.5 and 5 μM, the folds raised to 5.89 ± 2.33 and 6.27 ± 2.14 times respectively. Taken together, the observations imply that CuE significantly reduced the mitochondrial membrane potential of SAS cells.

Throughout Asia, CuE has been used in traditional medicine to treat tumors and many studies have attempted to elucidate the mechanism underlying its anticancer activity [13].

In this study, CuE demonstrated anticancer activity as well as the ability to induce apoptosis in SAS cells. The results collected in this series of studies provide experimental evidence supporting the contention that CuE may irreversibly arrest tumor cell growth. The results of mechanistic analysis led to the conclusion that both the inhibition of proliferation and the induction of cell cycle arrest are highly dependent upon CuE accumulated in the cancer cells.

Multiple apoptotic stimuli trigger the activation of proteases called caspases, which in turn initiate and execute the apoptotic program [14]. Caspases, a family of cysteinyl aspartate-specific proteases, play an essential role in the regulation and execution of apoptotic cell death. Multiple apoptotic stimuli trigger the activation of these proteases, which are constitutively expressed in nearly all cell types as inactive proenzymes [15]. All caspases are produced in cells as inactive zymogens, requiring a proteolytic cleavage and conversion to active form during apoptosis. Caspase-3 is the most extensively studied apoptotic protein and a key effector in the apoptosis pathway, amplifying the signal from initiator caspases (such as caspase-8) and signifying full commitment to cellular disassembly [16]. Activity and plasma membrane disintegration similar to that of caspase-3 serves as a measure of early apoptosis; whereas nuclear fragmentation serves as an indicator of late apoptosis [17]. These subunits then form an active protease capable of cleaving caspase-3, which initiates the death cascade and finally induction of apoptosis. Therefore, CuE-induced apoptosis is mediated through the activation of caspase 3.

## 3. Materials and Methods

### 3.1. Materials

Cucurbitacin E, DMSO (dimethyl sulfoxide) and MTT [3-(4,5-dimethylthiazol-2-yl)-2,5-diphenyltetrazolium bromide] were obtained from Sigma (St. Louis, MO, USA). Dulbecco Modified Eagle Medium (DMEM), fetal bovine serum, antibiotics, sodium pyruvate, trypsin, and phosphate-buffered saline (PBS) were purchased from Gibco, BRL (Grand Island, NY, USA). Polyvinylidene fluoride membrane (PVDF) (Millipore), and molecular weight markers were purchased from Bio Rad (Berkeley, CA, USA). All other reagents and compounds were analytical grades.

### 3.2. Cells

The SAS cells were maintained on culture dishes, in 90% (*v*/*v*) DMEM with 2 mM l-glutamine and contain 1.5 g/L sodium bicarbonate with 10% (*v*/*v*) fetal bovine serum (FBS). The cells were cultured in an atmosphere containing 5% CO_2_ in a 37 °C incubator.

### 3.3. Cell Proliferation Assay

The cells were seeded into 96-well culture plate at 5000 cells/well. The cells were treated with 0, 1.25, 2.5 and 5 μM CuE for 1 to 3 days. MTT dye (1 mg/mL) was added to each well for the at least 4 h of treatment. The reaction was stopped by the addition of DMSO, and optical density was measured at 540 nm on a multi-well plate reader. Background absorbance of the medium in the absence of cells was subtracted. All samples were assayed in triplicate, and the mean for each experiment was calculated. Results were expressed as a percentage of control, which was considered as 100%. Each assay was carried out in triplicate and the results were expressed as the mean (±SEM).

### 3.4. Measurement of Apoptosis

The SAS cells were first seeded in 6-well plates from Orange Scientific (Braine-l’Alleud, Belgium). Following treatment with CuE for 4 h, the cells were harvested. The cells were re-centrifuged (the supernatant discarded) and resuspended/incubated in 1× annexin-binding buffer. 5 μL of annexin V-FITC (BD Pharmingen, San Diego, CA, USA) and 1 μL of 100 μg/mL PI working solution for 15 min. Following the incubation period, the stained cells were analyzed using FACSCalibur flow cytometry (BD, Franklin Lakes, NJ, USA). Data was analyzed using WinMDI 2.8 free software (BD, Franklin Lakes, NJ, USA).

### 3.5. Caspase 3 Activity Assay

The caspase activity was assessed by the FITC rabbit anti-active caspase-3 (BD Pharmingen, San Diego, CA, USA). The cells were treated with CuE of 0, 1.25, 2.5 and 5 μM for 24 h. The caspase activity was detected and inspected by the FACSCalibur flow cytometry. Data was analyzed using WinMDI 2.8 free software (BD, Franklin Lakes, NJ, USA).

### 3.6. Cell Cycle Analysis

For cell cycle analysis we used the fluorescent nucleic acid dye propidium iodide (PI) to identify the proportion of cells in each of the three interphase stages of the cell cycle. Cells were treated with CuE for 24 h, and then harvested and fixed in 1 mL cold 70% ethanol for at least eight hours at −20 °C. DNA was stained in PI/RNaseA solution and the DNA content was detected using flow cytometry. Data was analyzed using WinMDI 2.8 free software.

### 3.7. Western Blot Assay

A total of 50 μg of proteins were separated by 10% SDS-PAGE, and transferred to PVDF membranes (Merck Millipore, Darmstadt, Germany). The membranes were blocked with blocking buffer (Odyddey, Lincoln, NE, USA) overnight, and incubated with anti-β-actin (Sigma-Aldrich, St. Louis, MO, USA), anti-caspase 3 (Santa Cruz BioTechnology, Dallas, TX, USA) antibodies for 1.5–2 h. The blots were washed and incubated with a second antibody (IRDye Li-COR, Lincoln, NE, USA) at a 1/20,000 dilution for 30 min. The antigen was then visualized using a near infrared imaging system (Odyssey LI-COR, Lincoln, NE, USA) and data was analyzed using Odyssey 2.1 software (Odyssey LI-COR, Lincoln, NE, USA).

### 3.8. Evaluation of Mitochondrial Membrane Potential (MMP; Δψ m)

The cells were first seeded in the 24-well plates (Orange, Hertfordshire, UK). Following the treatment with CuE for 4 h, JC-1 (10 μg/mL, Sigma, Ronkonkoma, NY, USA) was added to the culture medium, 50 μL per well, and then incubated (at 37 °C for 20 min) for mitochondria staining. After washing twice with a warm PBS, the cells were fixed with 2% paraformaldehyde, inspected by FACSCalibur flow cytometry. Data was analyzed using WinMDI 2.8 free software (BD, Franklin Lakes, NJ, USA).

### 3.9. Statistical Analysis

All data were reported as the mean (±SEM) of at least three separate experiments. A *t*-test or one-way ANOVA with post-hoc test was employed for statistical analysis, with significant differences determined as *p* < 0.05.

## 4. Conclusions

This study demonstrates for the first time that CuE is an effective inhibitor of OSCC tumors. The role of CuE in the inhibition of tumor growth was highlighted by the induction of apoptosis. Further study of this compound *in vivo* is necessary to understand the combined effects and therapeutic potential of chemotherapy drugs and CuE.
